# The DRESS trial: a feasibility randomized controlled trial of a neuropsychological approach to dressing therapy for stroke inpatients

**DOI:** 10.1177/0269215511431089

**Published:** 2012-08

**Authors:** Marion F Walker, Alan Sunderland, Joanna Fletcher-Smith, Avril Drummond, Pip Logan, Judi A Edmans, Katherine Garvey, Robert A Dineen, Paul Ince, Jane Horne, Rebecca J Fisher, Jenny L Taylor

**Affiliations:** 1 Division of Rehabilitation and Ageing, Community Health Sciences, University of Nottingham, UK; 2 School of Psychology, University of Nottingham, UK; 3 Division of Radiological and Imaging Sciences, School of Clinical Sciences, University of Nottingham, UK

**Keywords:** Stroke, rehabilitation, activities of daily living, cognitive impairment, occupational therapy

## Abstract

**Objective:**

To investigate two approaches to treating patients with persistent dressing problems and cognitive difficulties following stroke.

**Design:**

Pilot randomized controlled trial.

**Setting:**

Inpatient stroke rehabilitation service.

**Subjects:**

Seventy consecutive stroke patients with persistent dressing problems and accompanying cognitive difficulties at two weeks after their stroke.

**Interventions:**

Patients were randomly allocated to six weeks of either a systematic neuropsychological approach, based on analysis of dressing problems and further cognitive testing, or to the control group who received conventional (functional) dressing practice. Both groups received treatment three times a week in accordance with two separately prepared manuals.

**Main measures:**

Nottingham Stroke Dressing Assessment (NSDA), Line Cancellation, 10-hole peg transfer test, Object Decision, Gesture Imitation. Patients were assessed at six weeks after randomization by an independent assessor masked to group allocation.

**Results:**

Both neuropsychological and functional groups improved performance on the NSDA over the treatment period (31% and 22%, respectively) but there was no significant difference between groups at six weeks. However, the neuropsychological group showed a significantly greater improvement on a line cancellation test of visual neglect (*t*(62) = 2.1, *P* < 0.05) and a planned subanalysis for those with right hemisphere damage showed a trend towards better dressing outcome (*P* = 0.07, one-tailed).

**Conclusions:**

Results demonstrate the potential benefits of a systematic neuropsychological approach to dressing therapy, particularly for patients with right hemisphere damage. This study suggests the need for a phase III study evaluating the efficacy of a systematic neuropsychological approach in treating dressing difficulties, targeting patients with right hemisphere stroke and visuospatial impairments.

## Introduction

Dressing is a daily activity which is taken for granted by the able-bodied. Following stroke this self-care task can be problematic, with 54% of stroke survivors unable to dress independently at six months^1^ and 36% at two years after stroke.^2^ The prevalence of this problem is unsurprising, however, as dressing is a complex skill that requires many physical and cognitive abilities to ensure independence.^3^ Previous longitudinal studies have documented that those patients with persistent cognitive difficulties have higher levels of dressing dependence than those without^2^ and that the nature of cognitive difficulties determines the pattern of persistent dressing problems.^4^

As part of routine stroke rehabilitation, occupational therapists assess the self-care abilities of each patient and strive to resolve any dressing difficulties observed. A narrative literature review^5^ and survey of occupational therapy dressing practices in the UK^6^ documented that therapists did not use standardized dressing assessments to evaluate dressing performance nor did they use research evidence to inform their clinical practice, frequently providing a time-limited, repetitive, problem-solving approach to dressing practice. This method is referred to as the ‘functional approach’ to dressing. Although cognitive deficits were acknowledged as a key prohibitive factor in the acquisition of dressing independence, there was little evidence in the survey of therapists tailoring the approach to dressing treatment in light of impairments experienced by the patient. Therapists who did treat cognitive impairments did so using mental stimulation on unrelated cognitive exercises, such as pen and paper exercises to improve visual neglect, in the hope that improvements on these tasks might generalize to dressing ability. There was little evidence of therapists treating the cognitive difficulties directly during dressing practice.

There is some evidence that dressing practice provided by occupational therapists can be beneficial. A previous single-blind, randomized cross-over trial has described the successful treatment of dressing difficulties after stroke in a group of community-dwelling patients (*n =* 30) at six months after stroke.^7^ Dressing practice was administered by an experienced occupational therapist over a three-month period and employed a pragmatic functional approach to treatment. The interventions provided included advice on appropriate clothing, the teaching of strategies such as dressing the affected side first, the use of markers on garments to overcome perceptual problems and energy conservation techniques. Although there was an average increase in dressing ability (measured by the Nottingham Stroke Dressing Assessment^3^) of 11% in the treatment group, it is possible that an optimal improvement in dressing ability was not achieved as there was no systematic approach to the assessment, analysis of the underlying problems or targeted treatment of cognitive difficulties. Similarly, although two other studies have reported improvements in dressing performance following task-specific interventions,^8,9^ the underlying cognitive impairments associated with dressing remain unexplored.

A subsequent single-blind randomized multiple-baseline experiment^10^ combined naturalistic observation of dressing abilities, systematic neuropsychological assessment and administration of targeted dressing interventions. Results demonstrated that there was a significant treatment effect, measured by the Nottingham Stroke Dressing Assessment and observation-based t-shirt test, for those inpatients with right hemisphere stroke. There was, however, no therapy-related improvement for those with left or bilateral damage and apraxia. Because of the small number of case studies in this experiment (*n =* 8), a further study was required to establish the potential benefits of this approach over conventional approaches employed by occupational therapists. In following the MRC framework for the development and evaluation of complex interventions^11^ a pilot phase II randomized controlled trial was designed. Our aim was to conduct an evaluation of two approaches used to treat stroke patients with persistent dressing problems and accompanying cognitive difficulties after stroke.

## Methods

Consecutive inpatients on the stroke rehabilitation wards at Nottingham University Hospitals Trust were monitored by the ward occupational therapists to identify those patients with persistent dressing difficulties. Patients were deemed suitable for the study if they had received two weeks of conventional rehabilitation and still required help to dress. Patients were invited to take part in the study if they were impaired (scored less than 100% maximum score) on the Nottingham Stroke Dressing Assessment^3,12^ and on one or more items in a brief cognitive screening test: line cancellation^13^ to detect visual neglect (maximum score 36, impaired <33); 10-hole peg transfer test^14^ with the non-paretic hand to detect dexterity problems not due to paresis (impaired >22 seconds); the Object Decision subtest from the Visual Object and Space Perception assessment^15^ (maximum score 20, impaired <12); Gesture Imitation to detect apraxia^16^ (maximum score 20, impaired <15). To ensure selection of patients able to participate in dressing practice, the exclusion criteria included the inability to tolerate sitting in a chair for 15 minutes, premorbid disability (Rankin^17^ >3) and known diagnosis of depression or dementia.

In terms of comprehension, patients had to be able to understand English if it wasn’t their first language. The Sheffield Aphasia Test^18^ was used to assess aphasia and adapted information and consent forms were used where appropriate. Demographic data were also collected on the Barthel Index,^19^ Motricity Index,^20^ age, gender and side of stroke.

Following baseline assessments and using concealed allocation via the University of Nottingham Clinical Trials Unit internet randomization service, patients were randomized to one of two treatment groups; conventional occupational therapy (the ‘Functional approach’) or the ‘Neuropsychological approach.’ Patients were stratified by side of stroke and severity of their dressing problem as measured by the Nottingham Stroke Dressing Assessment score. The two groups continued with their usual rehabilitation therapy and nursing care and only differed in the type of dressing practice provided by the trial occupational therapists. Both interventions were delivered by two research occupational therapists experienced in the treatment of stroke patients.

As side of stroke was initially recorded from the medical notes, brain scans were later reviewed by an experienced stroke radiologist. This identified a subgroup of patients with definite or probable bilateral hemisphere damage and these were treated as a separate category in analysis of results.

Interventions were prescribed according to group allocation. Treatment manuals had previously been developed for both dressing approaches using comprehensive literature searches, survey results^6^ and occupational therapy text books.^21^ Patients allocated to the functional approach were given repeated dressing practice using a problem-solving approach, with assistance when required. This approach is commonly used by occupational therapists in the UK and has been shown to have a beneficial effect on dressing performance.^7^ Dressing interventions would include components such as putting the affected arm into the sleeve first, crossing affected leg over other leg to reach feet, energy conservation techniques, etc. There was no attempt to formally assess the patient’s cognitive difficulties or relate them to evidence on which approach to training might be the most successful.

Patients assigned to the neuropsychological approach received further detailed cognitive testing and an assessment of the impact of cognitive deficits on dressing by observation of a standard task of putting on a t-shirt,^10^ with performance scored using an error analysis rating form.^12^ This error analysis identified the presence of cognitive problems such as impaired attention, spatial confusion and action sequence errors. On the basis of the test results and the types of error observed, treatment interventions were selected from a menu of evidence-based techniques described in the pre-prepared neuropsychological treatment manual. The most commonly used specific techniques were cueing and alerting procedures to combat neglect or attentional difficulties,^22–24^ systematic laying out of clothing to reduce spatial confusion,^10^ and graded errorless learning strategies to enhance acquisition of dressing skills.^25^ Fidelity of treatment in both patient groups was monitored by an independent researcher who observed random dressing sessions to ensure the manuals were adhered to.

The optimal intensity of dressing practice as indicated in our previous single case experiments^10^ dictated that patients in both groups received dressing treatment three times a week for a period of six weeks. Patients continued to receive dressing treatment in their own home if they were discharged from hospital before the end of the treatment period.

Patients were assessed at six weeks after randomization by an independent assessor who was masked to the patient’s treatment group allocation. Masking of the independent assessor was monitored by completion of a best guess form. All patients were assessed on the Nottingham Stroke Dressing Assessment^3,12^ and the cognitive tests which had been used in initial screening were repeated (line cancellation^13^ to detect visual neglect; 10-hole peg transfer test^14^ with the non-paretic hand to detect dexterity problems not due to paresis; the Object Decision subtest from the Visual Object and Space Perception assessment;^15^ Gesture Imitation to detect apraxia^16^). Performance on the Nottingham Stroke Dressing Assessment was selected as the primary outcome measure for the study.

As this was a feasibility study, no formal power calculation was performed. However based on a similar dressing study^7^ using the same primary outcome measure, it was estimated that the study would require 35 patients per group (80% power to detect an effect at the 0.05 level). Although the previous trial had investigated a slightly different approach to dressing difficulties, the authors felt that a pragmatic study of this magnitude would be informative for the proposed trial. Statistical analyses included Student’s *t*-tests of within group means and standard deviations over time, between group differences at six weeks and planned subgroup analyses for patients with right hemisphere and those with left or bilateral damage were also carried out.

## Results

Of the 965 patients screened during the trial (1 March 2008–28 February 2010), we sought consent from 110. Of these, 40 passed the screening tests. The remaining 70 patients (64%) were randomized to either the neuropsychological group (*n =* 36) or to the functional group (*n =* 34). A sample of treatment sessions were monitored to ensure that they included the actual treatment prescribed in the manual. We found a high level of fidelity of treatment in both treatment groups. Masking of the outcome assessor was tested and found to be compromised for only six patients.

**Figure 1.**
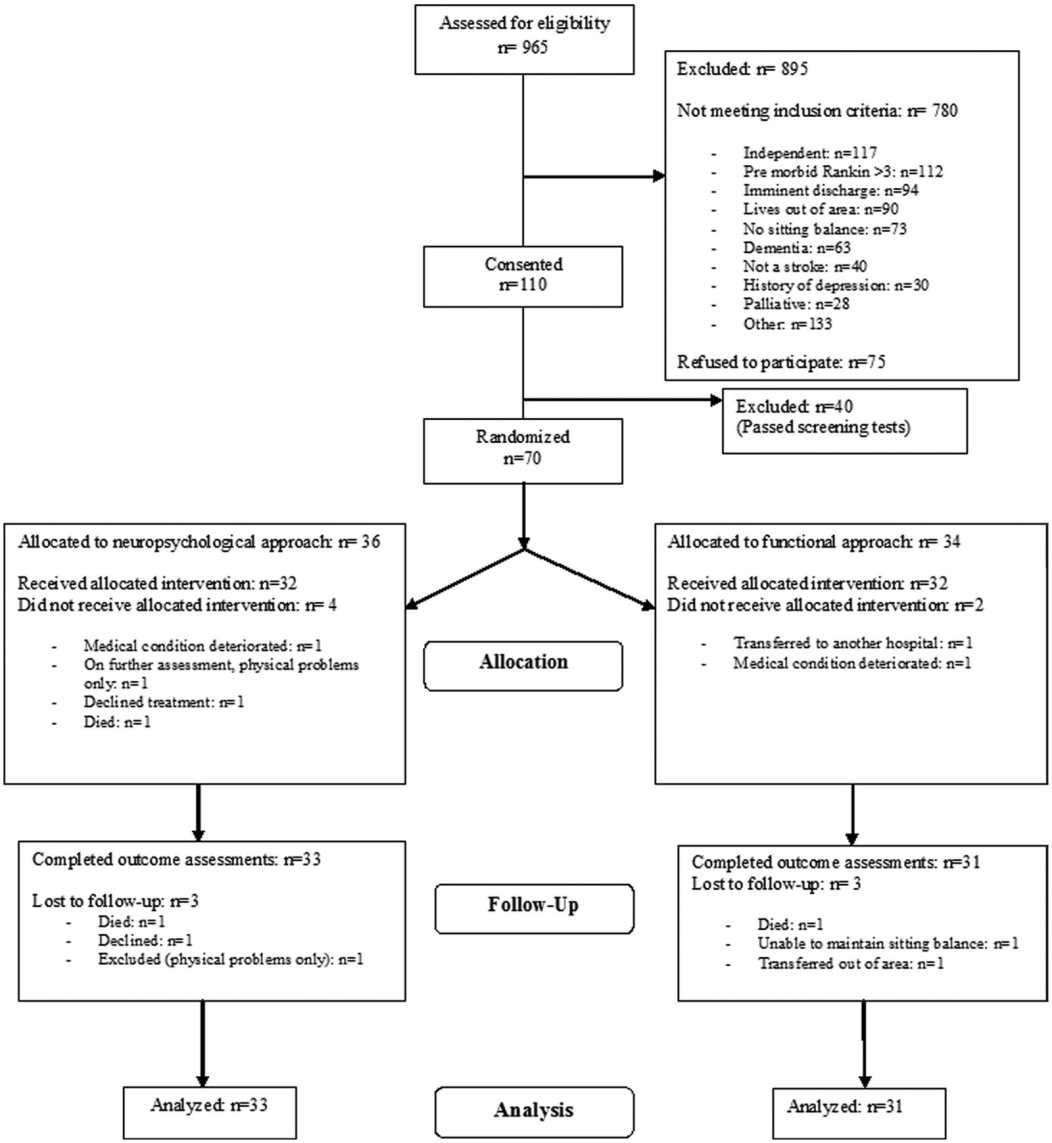
Dressing Rehabilitation Evaluation Stroke Study (DRESS).

Figure 1 shows patient selection and drop-outs.

Table 1 shows the details of those who completed the trial and Table 2 shows their scores on the baseline assessments. The treatment groups were well matched on all variables.

**Table 1.** Background details of patients who completed the trial

	Neuropsychological group (*N* = 33)	Functional group (*N* = 31)
Years of age.		
Median	77	81
IQR	73–83	74–84
Range	47–93	41–96
Days since stroke.		
Median	26	22
IQR	19–40	18–33
Range	12–139	13–99
Sex		
Female	21	17
Male	12	14
Site of brain lesions		
Left hemisphere	13	6
Right hemisphere	14	15
Bilateral or brainstem	6	10

**Table 2.** Scores on baseline assessments

	Neuropsychological group (*N* = 33)	Functional group (*N* = 31)
NSDA %		
Mean (SD)	37 (31)	42 (30)
Range	0–95	0–92
Barthel ADL (max = 20)		
Mean (SD)	6.4 (3.6)	6.7 (4.6)
Range	2–16	1–17
Motricity Index (max = 100)		
Mean (SD)	53 (30)	49 (33)
Range	0–100	1–17
Sheffield Aphasia Test		
Mean (SD)	12 (6)	13 (5)
% Impaired (<15)	50%	36%
**Cognitive Screening Tests**		
Line Cancellation		
Mean (SD)	23 (14)	27 (11)
% Impaired (<33)	48%	48%
Object Decision		
Mean (SD)	11 (4)	11 (3)
% Impaired (<12)	51%	51%
Pegs per second		
Mean (SD)	0.47 (0.21)	0.44 (0.18)
% Impaired (<0.45)	41%	45%
Gesture Imitation		
Mean (SD)	15 (4)	16 (3)
% Impaired (<15)	27%	16%

NSDA, Nottingham Stroke Dressing Assessment. This was the primary outcome measure.

The interventions provided in both arms of the study were well tolerated and found to be acceptable to patients. (Due to space limitations these findings on acceptability will be reported in detail in a further paper.) The number of treatment sessions delivered to each group during the six-week period was well matched. The Neuropsychological group received a median of 13 sessions (min 0, max 18) and the Functional group received a median number of 12 sessions (min 0, max 18). The key reasons for people not receiving all 18 sessions were: the patient deteriorated or died, moved out of the geographical catchment area, nursing staff had dressed the patient before the therapist arrived or the patient had reached independent dressing before the end of the six-week intervention period.

Performance at the outcome assessments is shown in Table 3. Compared to the baseline assessments, both treatment groups showed significant improvements in dressing ability (improvements of 31% and 22% on the Nottingham Stroke Dressing Assessment for the Neuropsychological and Functional groups respectively) but the groups did not differ significantly in this respect (*t*(62) = 1.3, NS).

**Table 3.** Mean (SD) scores at baseline and 6 weeks for primary outcome measure (NSDA), and secondary cognitive subtests

	NP group (*N* = 33)	Functional group (*N* = 31)	Mean change (SD) from baseline at 6 weeks		Mean advantage for NP group (95% CI)
			NP group	Functional group	
NSDA (%)	69 (35)	65 (32)	31** (31)	22** (17)	9 (–4 to 21)
Cognitive tests					
Line Cancellation	29 (11)	27 (11)	5.5** (9.8)	–0.5 (12.5)	6.0* (0.4 to 11)
Object Decision	12 (5.7)	12 (5.6)	1.4 (4.2)	1.6* (4.4)	–0.2 (–2.4 to 1.9)
Pegs per second	0.56 (0.26)	0.58 (0.18)	0.11** (0.13)	0.12** (0.17)	–0.01 (–0.09 to 0.06)
Gesture Imitation	17 (4.9)	17 (3.0)	1.6* (3.7)	0.9 (2.9)	0.7 (–0.9 to 2.4)

*t*-tests, *P < 0.05 **P < 0.01 two-tailed. All other changes NS.

NP, Neuropsychological; NSDA, Nottingham Stroke Dressing Assessment; CI, confidence interval.

Table 3 also demonstrates that the cognitive tests showed a trend towards improvement from baseline. The Neuropsychological group showed a greater reduction in visual neglect on the cancellation test than the Functional group (*t*(62) = 2.1, *P* < 0.05). There were no significant group differences on any other cognitive test.

**Figure 2.**
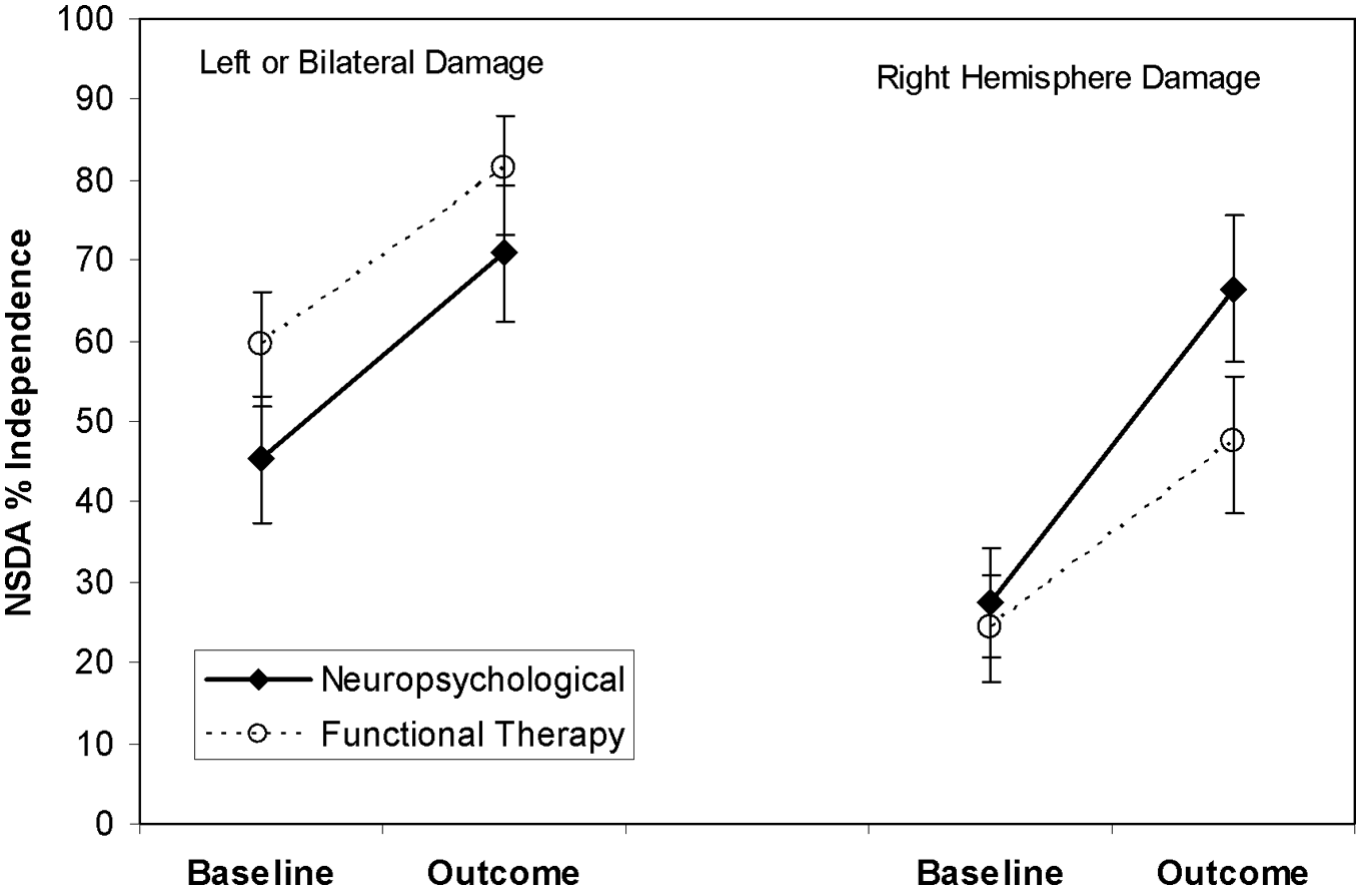
Mean scores on the Nottingham Stroke Dressing Assessment for the patient subgroups at baseline and at six weeks follow-up. The error bars show the standard error of the mean. NSDA, Nottingham Stroke Dressing Assessment.

Following the approach used in our previous single-case design investigation,^8^ Table 4 and Figure 2 show planned subgroup analyses for patients with right hemisphere and those with left or bilateral damage. These show a trend towards greater dressing improvement with the neuropsychological approach for those with right hemisphere damage (*P* = 0.07, one-tailed) who also show a significantly greater reduction of visual neglect on the line cancellation test. In contrast, the subgroup with left or bilateral damage show no differences between treatment approaches close to significance, either in dressing or test performance.

**Table 4.** Mean (SD) scores at baseline and six weeks for NSDA, and secondary cognitive subtests. Planned subgroup analysis

	Left or bilateral lesions				Advantage for NP group (CI)	Right hemisphere lesions				Advantage for NP group (CI)
	Baseline scores		Change from baseline			Baseline scores		Change from baseline		
		
	NP group (*N* = 19)	Functional group (*N* = 16)	NP group	Functional group		NP group (*N* = 14)	Functional group (*N* = 15)	NP group	Functional group	
NSDA %	70 (37)	81 (25)	25**	21**	4 (12 to 20)	66 (33)	47 (31)	39**	23**	16 (–5 to 36)
Cognitive Tests										
Line Cancellation	27 (13)	29 (12)	2.8	–1.6	4.4 (–4 to 13)	33 (7.1)	25 (11)	9.2*	0.8	8.4* (1.1 to 15.5)
Object Decision	12 (6.4)	14 (6.1)	–0.1	1.5	–1.6 (–4.9 to 1.5)	13 (4.8)	11 (5.0)	3.5**	1.7	1.8 (–0.9 to 4.3)
Pegs per second	0.54 (0.29)	0.55 (0.17)	0.08*	0.05	0.03 (–0.08 to 0.14)	0.57 (0.21)	0.61 (0.18)	0.14**	0.19**	–0.05 (–0.15 to 0.04)
Gesture Imitation	16 (6.2)	16 (3.8)	2.1	1.1	1.0 (–2.0 to 2.1)	19 (0.7)	19 (1.1)	1.1	0.6	0.5 (–1.0 to 2.0)

*t*-tests, *P < 0.05 **P < 0.01 two-tailed. All other changes NS.

NP, Neuropsychological; NSDA, Nottingham Stroke Dressing Assessment; CI, confidence interval.

## Discussion

Our feasibility randomized controlled trial indicates that both groups improved in their dressing performance over the trial intervention period. We found no statistically significant differences between the groups on performance on our main outcome measure: the Nottingham Stroke Dressing Assessment. However we did find a trend for improvement in those patients with right hemisphere stroke who were receiving the systematic neuropsychological approach to dressing. This outcome supports our previous findings in this patient group^10^ and adds weight to the hypothesis that patients with right hemisphere stroke with accompanying dressing problems and cognitive deficits can be treated successfully using a systematic neuropsychological approach. The fact that these patients also demonstrated a significantly greater reduction in visual neglect suggest that it was the use of techniques to reduce neglect which had the greatest impact on dressing ability in this group.

It is likely that a stronger treatment effect would be observed in a purpose designed trial aimed specifically at patients with right hemisphere damage. The current study included all patients with cognitive impairment and dressing difficulties, with no preselection for site of brain lesion. This meant that the subgroup of right hemisphere patients allocated to the neuropsychological was small. Furthermore, the techniques described in our treatment manual were culled from the wider neuropsychological literature and seldom specifically described the application of these treatments to patients with dressing difficulties.^22–25^ Our experience of the trial will allow us to write a more targeted treatment manual, which together with a larger sample size may demonstrate a more substantial treatment effect. In addition, we included two intervention groups in our dressing trial and did not include a conventional control group. Our reasoning behind the inclusion of a manualized functional approach was based on the positive benefits found in a similar interventional dressing study.^7^ Although the approach we applied was based on routine care provided by occupational therapists in the UK, the content of the functional manual was very prescriptive and the frequency with which the intervention was delivered was much greater than that routinely provided on the stroke rehabilitation wards. We therefore believe that a clinically significant difference may have been observed on the main outcome measure had we included a conventional control group.

The left and bilateral damage subgroup showed no sign of any benefit of the neuropsychological approach. One reason for this may be that their dressing difficulties were simply less severe and therefore that there was less room for improvement. For right hemisphere-damaged patients, the presence of unilateral neglect, spatial confusion or poor sustained attention have a devastating effect on dressing skills.^3^ In contrast, left hemisphere damage is associated with apraxia and related problems in control of action, but the impact of these on everyday functioning is more subtle and seems to depend on the exact cognitive demands of any given task situation.^26^ This said, there were left hemisphere damaged apraxic patients in this study who were unable to regain independence in dressing, and reasons for this lack of benefit of the neuropsychological approach need to be considered. A likely explanation is that the techniques to assist them are not well developed: there are only few, small-scale experimental studies on intervention for apraxia.^27^ This contrasts with the well-developed literature on intervention for visual neglect.^28^ Our results suggest that evidence-based systematic interventions tailored to the deficits found in right hemisphere-damaged stroke patients are likely to be beneficial.

The main weakness of our study was its relatively small sample size which limited the power to detect a statistically significant effect on dressing performance. Similarly, although the subgroup analysis was planned, it was nonetheless carried out on a small number of patients. However, we believe that the sample size achieved has allowed us to demonstrate that this approach to dressing is a feasible method to employ with stroke patients experiencing persistent dressing difficulties and such an intervention can be carried out on a busy stroke rehabilitation unit. We believe the indication of possible benefit in right hemisphere stroke is worthy of further enquiry and should be tested in a multicentre trial utilizing the findings from this study.

**Clinical messages**

- A systematic assessment using a standardized dressing assessment and neuropsychological assessments can be helpful in identifying the cause of persistent dressing difficulties after stroke.
- A neuropsychological approach to the treatment of persistent dressing difficulties may be beneficial for stroke patients with cognitive difficulties.
